# High Frequency of Multidrug-Resistant (MDR) Atypical Enteropathogenic *Escherichia coli* (aEPEC) in Broilers in Hungary

**DOI:** 10.3389/fvets.2020.00511

**Published:** 2020-08-13

**Authors:** András Adorján, László Makrai, Tünde Mag, Szilárd Jánosi, László Könyves, István Tóth

**Affiliations:** ^1^Department of Microbiology and Infectious Diseases, University of Veterinary Medicine, Budapest, Hungary; ^2^National Public Health Center, Budapest, Hungary; ^3^Veterinary Diagnostic Directorate, National Food Chain Safety Office, Budapest, Hungary; ^4^Department of Animal Hygiene and Mobile Clinic, University of Veterinary Medicine, Budapest, Hungary; ^5^Institute for Veterinary Medical Research, Agricultural Research Center, Budapest, Hungary

**Keywords:** *Escherichia coli*, atypical enteropathogenic *Escherichia coli* (EPEC), poultry, multidrug resistance, zoonosis

## Abstract

*Escherichia coli* (EC) strains belong to several pathotypes capable of infecting both humans and animals. Some of them have zoonotic potential and can sporadically cause epidemic outbreaks. Our aim was to screen for the distribution of these pathotypes in broilers and their related products. Therefore, *E. coli* strains were isolated (*n* = 118) from poultry intestine (*n* = 57), carcass (*n* = 57), and wastewater (*n* = 4) samples from one slaughterhouse with own reared poultry source and the National Reference Laboratory (NRL) poultry *E. coli* collection (*n* = 170) from the year 2017 was also studied. All 288 *E. coli* strains were screened by PCR for pathotype-specific genes *stx, eae, st-lt, aggR, ipaH*, and for further EPEC-specific virulence genes (*bfp, EAF, tir, perA, ler*). Altogether 35 atypical enteropathogenic *E. coli* (aEPEC) strains from the slaughterhouse and 48 aEPEC strains from the NRL collection were found. Regarding the phylogenetic groups of aEPEC, all four main groups were represented but there was a shift toward the B2 group (25%) as compared with the non-EPEC isolates (3%). The aEPEC isolates belonged to serogroups O14, O108, and O45. Multidrug resistance (MDR) was abundant in aEPEC strains (80 out of 83 aEPEC) with a diverse resistance pattern (*n* = 56). Our results of this study indicate that the high frequency of aEPEC in broilers and on their carcass surface, with frequent MDR to several antibiotic groups, raises the possibility that these strains pose a zoonotic risk to humans.

## Introduction

*Escherichia coli* is a member of the normal gut microbiota of several animal species including poultry. Commensal *E. coli* strains play a role in maintaining the normal gut microbiota. However, some of them carry virulence genes and can cause mostly extraintestinal infections in birds (avian pathogenic strains, APEC) or have zoonotic potential. These strains may also be transmitted to humans through the food chain (1–4). The avian *E. coli* strains can also harbor antibiotic resistance genes encoding resistance to antimicrobials of human therapeutic significance (5, 6).

Potentially zoonotic *E. coli* strains can be categorized into two major groups: extraintestinal (ExPEC) and intestinal/diarrhoeagenic (DEC). The DEC strains differ from each other in terms of pathogenesis and form six well-described categories: enteropathogenic *E. coli* (EPEC), enterohaemorrhagic *E. coli* (EHEC), enterotoxigenic *E. coli* (ETEC), enteroaggregative *E. coli* (EAEC), enteroinvasive *E. coli* (EIEC), and diffusely adherent *E. coli* (DAEC) (6).

Although the aforementioned six pathotypes are mainly human pathogens and cause sporadic infections or outbreaks [for example the EHEC outbreak in Germany (7) or atypical EPEC (aEPEC) outbreaks in developing countries (8)], they are frequently isolated from animals (9–13). Poultry can also act as a reservoir of four pathotypes (EHEC, ETEC, EPEC, EAEC) with various distribution patterns and incidence rates on poultry-related products (11, 14, 15) or in their intestinal microbiota (11, 16, 17), and increase the risk of their zoonotic potential (18).

In the present study we examined the occurrence of DEC in poultry intestine and in poultry-related products. The phylogroups and serogroups of the DEC isolates and their antibiotic resistance were also studied. The poultry *E. coli* collection of the National Reference Laboratory (NRL) from the same year was also investigated. Our study revealed a high frequency of multidrug-resistant aEPEC in broilers in Hungary.

## Materials and Methods

### Collection of Samples and Isolation of *Escherichia coli*

Altogether 118 samples were collected from healthy poultries and four samples from slaughterhouse wastewater. These samples were taken from one slaughterhouse (SH) one occasion in each year in the years 2016 (*n* = 22) and 2017 (*n* = 96) where they slaughter broiler only from their own poultry farm. The samples included cotton swab samples from the ceacal content (*n* = 57) and from the inner surface of the carcass of slaughtered chickens (*n* = 57). Wastewater samples (*n* = 4) were also collected from the slaughterhouse effluent during its middle flow (2 ml for each sample). All samples were stored at +4°C at most for 2 h until further laboratory procedure. For prolonged storage the samples were deep frozen at −70°C.

For the isolation of *E. coli* strains bromothymol blue (BTB) selective agar plates were used. In each sample one characteristic lactose-fermenting coliform colonies were further purified on BTB and subjected to species identification by MALDI-TOF mass spectrometry (19, 20).

As reference, the representative collection of *E. coli* strains (*n* = 170) isolated from ceacal content of slaughtered healthy poultry by the NRL (National Food Chain Safety Office, Hungary) in 2017 was used for comparison. This collection represented 113 broiler farms from 16 slaughterhouses in 6 counties of Hungary.

### Phenotypic Methods

Serotyping was carried out by using O-specific immune sera as described by Ørskov et al. (21) at the National Center of Epidemiology, Hungary.

Colicin production was tested as described by Abbot et al. (22), using an *E. coli* K-12 strain sensitive to a wide range of colicin.

Antibiotic resistance was examined by the disk diffusion method on Mueller-Hinton agar against 15 antimicrobials from 10 groups, namely penicillins [ampicillin (10 μg)]; ß-lactams [amoxicillin (10 μg)]; cephems [cefotaxime (30 μg)]; aminoglycosides [gentamicin (10 μg), kanamycin (30 μg), streptomycin (10 μg)]; tetracyclines [tetracycline (30 μg)]; fluoroquinolones [ciprofloxacin (5 μg), enrofloxacin (5 μg)]; quinolones [nalidixic acid (30 μg)]; folate pathway inhibitors [trimethoprim (30 μg), sulphonamide (300 μg), trimethoprim + sulphonamide (1.25 μg/23.75 μg)]; phenicols [chloramphenicol (30 μg)]; nitrofurans [nitrofurantoin (300 μg)]. The standard evaluation protocol (M100-S25) of the Clinical and Laboratory Standards Institute (33) was followed throughout the procedure. The isolates were classified by the recorded zone diameter into a sensitive or a resistant group.

### DNA Methods

#### Genotypic Characterization of Isolates

Each isolated *Escherichia coli* was inoculated into 2 ml LB (Luria-Bertani) broth and incubated overnight at 37°C. DNA templates were performed with boiling method from these cultures. The pellets of 100 μl bacterial cultures were resuspended in 100 μl distilled water and boiled for 10 min. These suspensions were sedimented by centrifugation and the supernatants were used as template for PCR reaction.

*Escherichia coli* isolates were screened for the presence of virulence genes by PCR using published pathotype-specific primers and for detecting the LEE-encoded regulator *ler* gene which were designed in this study (with 60°C annealing temperature and amplicon size of 172 bp) by primer-BLAST (National Center of Biotechnology Information, U.S. National Library of Medicine). Details of used primers were summarized in Table 1.

**Table 1:** Used primers for PCR with their references.

**Genes**	**Primer names and sequences (5^**′**^-3^**′**^)**	**References**
*eae*	B52: AGGCTTCGTCACAGTTG	(23)
	B53: CCATCGTCACCAGAGGA	
*stx 1*	B54: AGAGCGATGTTACGGTTTG	(23)
	B55: TTGCCCCCAGAGTGGATG	
*stx 2*	B56: TGGGTTTTTCTTCGGTATC	(23)
	B57: GACATTCTGGTTGACTCTCTT	
*sta*	STa-F: TTTATTTCTGTATTGTCTTT	(24)
	STa-R: ATTACAACACAGTTCACAG	
*lt1*	LT1-F: AGCAGGTTTCCCACCGGATCACCA	(24)
	LT1-R: GTGCTCAGATTCTGGGTCTC	
*ipaH*	IPAH III: GTTCCTTGACCGCCTTTCCGATACCGTC	(25)
	IPAH IV: GCCGGTCAGCCACCCTCTGAGAGTAC	
*aggR*	aggR-3: CATCTCTTTGATAAGTCCTTCTCG	(26)
	aggRks-1: GTATACACAAAAGAAGGAAGC	
*bfpA*	EP1: AATGGTGCTTGCGCTTGCTGC	(27)
	EP2: GCCGCTTTATCCAACCTGGTA	
*eaf*	Eaf1: CAGGGTAAAAGAAAGATGATAA	(28)
	Eaf2: TATGGGGACCATGTATTATCA	
*perA*	K1693: CCCAAGCTTTGGCAATGTTCCTTGTGT	(29)
	perA-24F: AACAAACGCGCATGAAGGTG	
*tir*	tirY474-F: CATATTTATGATGAGGTCGCTC	(30)
	tirS478-F: TCTGTTCAGAATATGGGGAATA	
	tirR: TAAAAGTTCAGATCTTGATGACAT	
*ler*	ler-fw: GACCAGGTCTGCCCTTCTTC	designed in this study^a^
	ler-rev: GACTGCGAGAGCAGGAAGTT	
*mcr1*	CLR5F: CGGTCAGTCCGTTTGTTC	(31)
	CLR5R: CTTGGTCGGTCTGTAGGG	
*ChuA*	ChuA.1: GACGAACCAACGGTCAGGATACGGT	(32)
	CAGGAT	
	ChuA.2: TGCCGCCAGTACCAAAGACA	
*YjaA*	YjaA.1: TGAAGTGTCAGGAGACGCTG	(32)
	YjaA.2: ATGGAGAATGCGTTCCTCAAC	
*TspE4C2*	TspE4C2.1: GAGTAATGTCGGGGCATTCA	(32)
	TspE4C2.2: CGCGCCAACAAAGTATTACG	

a *Using BLAST (National Center of Biotechnology Information, U.S. National Library of Medicine)*.

#### Phylogenetic Classification

Phylogenetic groups of *E. coli* isolates were determined by multiplex PCR (*ChuA, YjaA, TspE4C2*) described by Clermont et al. (32).

### Statistical Analysis

The 95% confidence intervals of frequencies were determined by Epitools (Epitools epidemiological calculators) to calculate their estimated frequencies (34). The comparison of proportions were performed with Fischer's exact test (with 95% confidence intervals) by R statistical program (35).

## Results

### Bacterial Strains and Their Genetic Examination

Altogether 118 *E. coli* strains were isolated from 114 individual samples from poultry and 4 from slaughterhouse wastewater using MALDI-TOF identification. None of these *E. coli* isolates produced colicin. To explore the zoonotic potential of *E. coli* isolates, they were screened by PCR for the presence of pathotype-specific genes (*eae* for EPEC; *eae* and *stx* for EHEC; *aggR* for EAEC; *st, lt* for ETEC and *ipaH* for EIEC). A total of 35 strains (29.66%, 95% CI: 22.17–38.44%) from the SH (18 out of 57 from intestine, 16 out of 57 from carcass, 1 out of 4 from wastewater) and 48 strains (28.24%, 95% CI: 22.01–35.42%) from the NRL collection were proven to harbor the *eae* gene. No other pathotype-specific marker gene could be detected in the collection. The EPEC isolates uniformly harbored the tyrosine phosphorylated *tir* (30) and *ler* (LEE encoded regulator) genes (36). Further characterization of the EPEC poultry strains revealed that they are atypical EPEC (aEPEC) since the *bfp* and *perA* genes and their coding EAF plasmid were uniformly absent (37).

**Figure 1:**
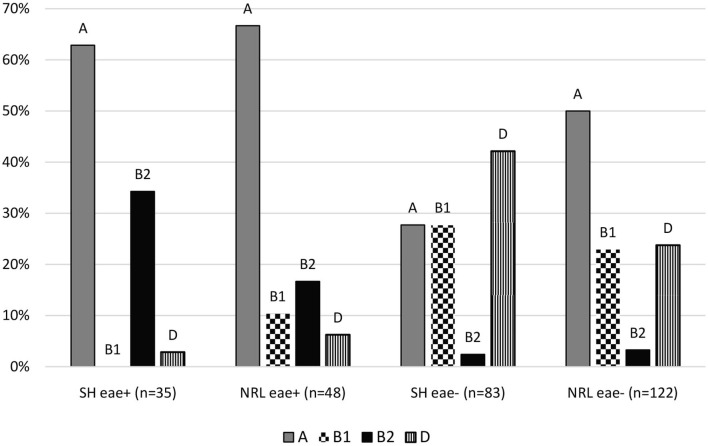
Distribution of the phylogenetic groups of isolates. SH, slaughterhouse; NRL, National Reference Laboratory; eae, intimin gene; A, B1, B2; D, main phylogenetic groups.

Non-EPEC and aEPEC strains differed in the composition of phylogenetic groups in each collection. *Escherichia coli* isolates from SH were belonging to A (28%, 95% CI: 19.23–38.16%), B1 (28%, 95% CI: 19.23–38.16%), B2 (2%, 95% CI: 0.66–8.37%), D (42%, 95% CI: 32.12–52.91%) phylogenetic groups as non-EPEC strains (*n* = 83) and A (63%, 95% CI: 46.34–76.83%), B1 (0%, 95% CI: 0.00–9.89%), B2 (34%, 95% CI: 20.83–50.85%), D (3%, 95% CI: 0.51–14.53%) phylogenetic groups as aEPEC strains (*n* = 35). Isolates from the NRL were belonging to A (50%, 95% CI: 41.26–58.74%), B1 (23%, 95% CI: 16.38–31.17%), B2 (3%, 95% CI: 1.28–8.13%), D (24%, 95% CI: 17.09–32.05%) phylogenetic groups as non-EPEC strains (*n* = 122) and A (67%, 95% CI: 52.54–78.32%), B1 (10%, 95% CI: 4.53–22.17%), B2 (17%, 95% CI: 8.70–29.58%), D (6%, 95% CI: 2.15–16.84%) phylogenetic groups as aEPEC strains (*n* = 48) (Figure 1).

We found significance (*p* ≤ 0.05) comparing phylogenetic groups of non-EPEC and aEPEC strains by Fischer's exact test except comparison of B1 and D phylogenetic groups, where the proportions are almost the same in the two groups. Therefore, these statistical results confirm that the pathogenicity of *Escherichia coli* has influence on the proportion of phylogenetic groups.

### Serogroups of aEPEC

The O antigen production of aEPEC strains from the SH was determined. The aEPEC isolates were found to belong to diverse serogroups, namely O14, O45, and O108. In samples from the year 2016, O108 was the dominant serogroup (10 out of 13), one O14 strain was identified in carcass, while two strains were non-typable (NT). Among strains from the year 2017, O14 was dominant (20 out of 22), one strain belonged to the O45 serogroup (wastewater) and one strain was NT (Table 2).

**Table 2:** Antibiotic resistance patterns of collected aEPEC strains with their origins, phylogenetic groups (ECOR) and O serogroups.

**Origin**	**Serogroup**	**ECOR**	**Resistance pattern**	***n* =**
**2016**				
Caecum	O108	B2	^a^NFT,SMX	1
Carcass	NT	A	^b^NFT,SMX	1
Caecum	O108	B2	^b^NAL,NFT,SMX	4
Carcass	O108	B2	^b^NAL,NFT,SMX	2
Caecum	O108	B2	CTX,NFT,SMX	2
Caecum	O108	B2	CTX,NAL,NFT,SMX	1
Caecum	NT	B2	CTX,GEN,NAL,NFT,SMX	1
Carcass	O14	A	AMP,CTX,NAL,NFT,SMX	1
**2017**				
Caecum	O14	A	CTX,GEN,KAN,NAL,SMX,STR	1
Carcass	O14	A	AMX,CTX,GEN,NAL,SMX,STR	1
Caecum	O14	A	^c^AMX,CTX,GEN,KAN,NAL,SMX,STR	2
Carcass	O14	A	^c^AMX,CTX,GEN,KAN,NAL,SMX,STR	2
Carcass	O14	A	AMP,CTX,KAN,NAL,SMX,STR	1
Caecum	O14	A	^d^AMP,CTX,GEN,KAN,NAL,SMX,STR	3
Carcass	O14	A	^d^AMP,CTX,GEN,KAN,NAL,SMX,STR	4
Sewage	O45	D	AMP,AMX,CTX,KAN,NAL,SMX,STR,SXT,TET,TMP	1
Carcass	O14	B2	AMP,AMX,CIP,CTX,ENR,GEN,NAL,SMX,STR,SXT,TMP	1
Caecum	NT	A	AMP,AMX,CTX,GEN,KAN,SMX,STR	1
Caecum	O14	A	^e^AMP,AMX,CTX,GEN,KAN,NAL,NFT,SMX,STR	1
Carcass	O14	A	^e^AMP,AMX,CTX,GEN,KAN,NAL,NFT,SMX,STR	2
Caecum	O14	A	^f^AMP,AMX,CTX,GEN,KAN,NAL,SMX,STR	1
Carcass	O14	A	^f^AMP,AMX,CTX,GEN,KAN,NAL,SMX,STR	1

*ECOR, phylogenetic group; ^a^same resistance pattern with different ECOR; ^b, c, d, e, f^have similar resistance pattern with different origin; NT, not typable; AMX, amoxicillin; AMP, ampicillin; CTX, cefotaxime; CIP, ciprofloxacin; ENR, enrofloxacin; GEN, gentamicin; KAN, kanamycin; CHL, chloramphenicol; NAL, nalidixic acid; NFT, nitrofurantoin; TET, tetracycline; STR, streptomycin; TMP, trimethoprim; SMX, sulphonamide; SXT, sulphonamide + trimethoprim*.

### Antibiotic Resistance

**Figure 2:**
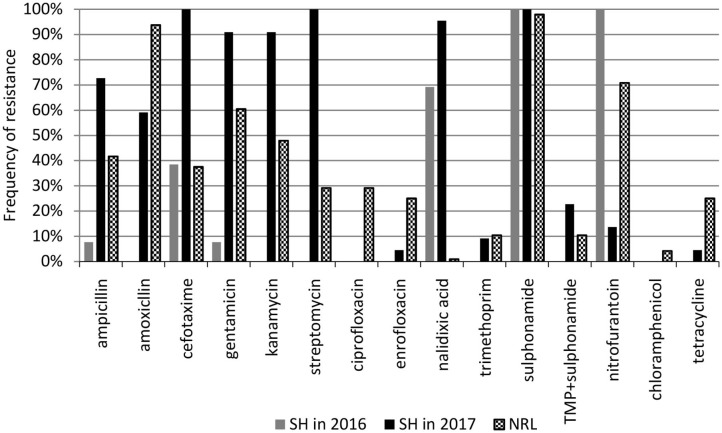
Frequency of antibiotic resistance among aEPEC strains. SH, slaughterhouse, NRL, National Reference Laboratory.

The antimicrobial sensitivity of poultry EPEC isolates was tested against 15 antibiotics representing 10 groups, as described in the Materials and methods. The frequency of antimicrobial resistance and the antibiotic resistance patterns are shown in Figure 2 and in Tables 2, 3, respectively.

**Table 3:** Antibiotic resistance patterns of aEPEC strains from the NRL collection with their phylogenetic groups (ECOR).

**Resistance pattern**	**ECOR**	***n* =**
NAL,SMX	A	1
GEN,NAL,NFT,SMX,TET	B2	1
AMX,NFT,SMX	A	1
AMX,NAL,NFT,SMX	A	1
AMX,KAN,NAL,SMX,STR,SXT,TMP	B1	1
AMX,GEN,NAL,SMX	A	1
AMX,GEN,NAL,NFT,SMX	B2	1
^d^AMX,GEN,KAN,NAL,NFT,SMX,TET	A	2
AMX,GEN,CTX,NFT,NAL,SMX,TET	A	1
AMX,CTX,SMX	D	1
^a^AMX,CTX,KAN,NAL,SMX	A	1
^a^AMX,CTX,KAN,NAL,SMX	B2	1
^d^AMX,CTX,KAN,NAL,NFT,SMX	A	4
AMX,CTX,GEN,KAN,NFT,SMX,STR,TET	A	1
AMX,CTX,GEN,KAN,NAL,NFT,SMX,STR	A	1
AMX,CIP,GEN,CTX,NFT,NAL,STR,SMX,SXT	B2	1
AMX,CIP,GEN,CTX,ENR,NFT,NAL,STR,SMX	A	1
AMX,CIP,ENR,NAL,NFT,SMX	A	1
AMX,CIP,ENR,KAN,NAL,SMX,TET	B2	1
AMX,CIP,CTX,GEN,NAL,NFT,SMX	A	1
AMX,CIP,CTX,ENR,NFT,NAL,STR,SMX	B2	1
AMX,CIP,CTX,ENR,KAN,NFT,SMX,STR,SXT,TET,TMP	A	1
AMX,CIP,CTX,ENR,GEN,KAN,NAL,NFT,SMX,STR	A	1
AMX,CIP,CTX,ENR,GEN,KAN,NAL,NFT,SMX	A	1
AMP,CHL,GEN,CIP,CTX,ENR,NFT,NAL,SMX,TET	A	1
AMP,AMX,NAL,SMX	A	1
AMP,AMX,KAN,NAL,SMX,STR	A	1
AMP,AMX,KAN,NAL,SMX	B2	1
AMP,AMX,GEN,NFT,KAN,SMX	A	1
AMP,AMX,GEN,KAN,NAL	A	1
AMP,AMX,GEN,CTX,NAL,STR,SMX	A	1
AMP,AMX,GEN,CIP,CTX,ENR,NFT,NAL,SMX,TMP,TET	D	1
AMP,AMX,CTX,NFT,NAL,STR,SMX,TET	A	1
AMP,AMX,CTX,NFT,NAL,STR,SMX	B1	1
^b^AMP,AMX,CTX,KAN,NAL,SMX	A	1
^b^AMP,AMX,CTX,KAN,NAL,SMX	B1	1
^c^AMP,AMX,CTX,GEN,NAL,NFT,SMX	A	1
^c^AMP,AMX,CTX,GEN,NAL,NFT,SMX	B1	1
AMP,AMX,CTX,GEN,KAN,NFT,SMX,STR,SXT,TMP	B1	1
AMP,AMX,CTX,GEN,KAN,NFT,NAL,SMX	A	1
AMP,AMX,CIP,GEN,CTX,ENR,NFT,NAL,STR,SMX,SXT,TET,TMP	D	1
AMP,AMX,CIP,ENR,GEN,NAL,NFT,SMX	A	1
AMP,AMX,CIP,CTX,GEN,KAN,NAL,NFT,STR,SMX	B2	1
AMP,AMX,CHL,ENR,GEN,KAN,NAL,SMX,STR,TET	A	1

*ECOR, phylogenetic group; ^a, b, c^same resistance pattern with different ECOR; ^d^have different origin of sample isolation; AMX, amoxicillin; AMP, ampicillin; CTX, cefotaxime; CIP, ciprofloxacin; ENR, enrofloxacin; GEN, gentamicin; KAN, kanamycin; CHL, chloramphenicol; NAL, nalidixic acid; NFT, nitrofurantoin; TET, tetracycline; STR, streptomycin; TMP, trimethoprim; SMX, sulphonamide; SXT, sulphonamide + trimethoprim*.

Frequency of antibiotic resistance among aEPEC (*n* = 35) originating from the SH changed between 2016 and 2017. In 2016, aEPECs (*n* = 13) were resistant to antibiotics belonging to 6 antibiotic groups, and all of them were resistant to sulphonamide (SMX) and nitrofurantoin (NFT). In 2017, aEPEC isolates (*n* = 22) were resistant to 9 antibiotic groups (except chloramphenicol), and all of them were resistant to cefotaxime (CTX), streptomycin (STR), and sulphonamide (SMX). Atypical EPEC strains from the NRL collection were resistant to 10 antibiotic groups where sulphonamide and amoxicillin resistance had the highest (98 and 94%, respectively), while nalidixic acid and chloramphenicol resistance had the lowest (1 and 4%, respectively) frequency (Figure 2).

Besides their wide variety of resistance, the aEPEC strains exhibited a high frequency of multidrug resistance (MDR) where they were resistant against at least three antibiotics. The overall frequency of MDR was 94 and 98% at the SH and in the NRL collection, respectively. The aEPEC strains showed 18 and 41 different resistance patterns at the SH (*n* = 35) and in the NRL collection (*n* = 48), respectively (Tables 2, 3).

None of the isolated strains had colistin resistance, as confirmed by the absence of the *mcr1* gene encoding colistin resistance (31).

## Discussion

In the present study, by investigating a total of 288 *Escherichia coli* strains isolated from poultry, 83 aEPEC strains were identified and characterized in Hungary. Interestingly, the distribution of pathotypes found in the samples differed from other published results. In previous studies, the main DEC pathotypes isolated from poultry and poultry products from retail markets were aEPEC, EHEC, ETEC, and EAEC (10, 11, 15–17); however, their frequency and ranking were found to be very variable (the frequency of aEPEC was between 2.3 and 56%). In our study the recorded frequency of aEPEC was around 30% (35 out of 118) in samples from the slaughterhouse and 28% (48 out of 170) in the NRL collection, and these values were in harmony with the frequency published earlier. Nonetheless, we could not identify any other pathotypes either from our 118 isolates or from the NRL collection. We suppose that this difference from the results of other authors is attributable to the fact that our samples originated directly from poultry, thus avoiding the possible cross-contamination from other meat or animal sources which can easily occur under retail market conditions. We presume that mixing of the meat bacteriota could occur during handling and alter the frequency of pathotypes found in retail market samples and in poultry-related products. The fact that cattle, sheep and pigs act as the main sources of EHEC and ETEC strains can further support this assumption (23, 38). Furthermore, we suppose that the isolation of *stx2*-positive *E. coli* from poultry in some previous studies (16, 39, 40) could be due to a possible contact with pigeons that frequently carry *stx2f*-positive *E. coli* (16, 41). Therefore, we think that poultry mainly carry aEPEC.

Investigation of slaughterhouse wastewater for the presence of any pathogenic *E. coli* strains, we also successfully identified an aEPEC strain (O45 serogroup and D phylogenetic group) from it. This finding reveals the possibility of other contamination sources in the SH than poultry, because it differs from other aEPEC phylogenetic and serogroups found in the same SH. Nonetheless, the contamination of wastewater proves that it can carry this pathogen from the slaughterhouse into the environment, thus posing an environmental hazard.

Investigating the genetic background, the capability of enterocyte effacement we found in all own isolated aEPEC strains that they possessed *ler* as LEE (Locus of enterocyte effacement) regulator gene and *tir* (translocated intimin receptor) beside carrying intimin gene. These two genes have major role in the process of enterocyte effacement by EPEC strains, especially in the adherence of intimin to the intestinal cell wall. Therefore, we suppose that these strains have capability to cause the characteristical enterocyte effacement.

Comparing the phylogenetic groups of aEPEC and non-EPEC strains, a shift toward the B2 group (24% comparing with 3%) and less B1 group (6% comparing with 25%) were seen for the aEPEC strains. B2 phylogenetic group has an importance because the ExPEC strains mainly belong to this group which strains mean more risk for possible zoonotic infection. At the same time, our aEPEC strains were very heterogeneous as all the four main phylogenetic groups were represented in our samples. This is in harmony with earlier results (17, 42) and supports the assumption regarding the heterogeneity of aEPEC strains (8, 37). The serogroups of the aEPEC strains also proved to be diverse: three different groups (O14, O45, O108) were identified, none of which belonged to the commonly demonstrated aEPEC serogroups (42). This finding further supports the notion that aEPEC strains are variable. However, each of the broiler flocks sampled yielded almost single and uniform serogroup.

Unfortunately, there was no information about the used antibiotics of sampled farms in years 2016 and 2017. However, the existence and increase of antibiotic resistance poses a major problem in both public health and veterinary practice. The antimicrobial resistance results obtained in this study are in harmony with the previously reported high frequency of resistance in aEPEC isolates (40, 43–47). Only 2 out of our 35 aEPEC isolates and 1 out of the 48 aEPEC strains in the NRL collection were not multidrug resistant. The remainder of the aEPEC isolates were resistant to several antibiotic groups and showed highly diverse antimicrobial resistance patterns, even in birds within the same flock. This poses the potential risk of spread of antimicrobial resistance to humans via their contact with poultry (48). It may also cause the lack of efficacy of antibiotic treatments performed in the everyday veterinary practice, because of gene shifting with the translocation of resistance genes among bacteria. The correlations found between aEPEC co-infections and the severity and outcome of diarrhea in animals (12, 49) further increase the importance of the high frequency of MDR found by us in aEPEC strains, as they might serve as a reservoir of resistance genes for other bacteria.

The results of this study indicate that the high frequency of aEPEC in intensively reared broilers and on their carcass surface, with frequent MDR to several antibiotic groups, raises the possibility that these strains pose a zoonotic risk to humans. Furthermore, it could be the reason the emergence of new multidrug-resistant bacteria in the last few years as well.

## Data Availability Statement

The raw data supporting the conclusions of this article will be made available by the authors, without undue reservation, to any qualified researcher.

## Author Contributions

AA performed most of the steps of experimental work. LM and SJ took part in the sample collection and their isolation. TM evaluated the O serogroups of the isolates. LK and IT took part in the coordination of experimental work and all of them participated in the writing of this scientific paper. All authors contributed to the article and approved the submitted version.

## Conflict of Interest

The authors declare that the research was conducted in the absence of any commercial or financial relationships that could be construed as a potential conflict of interest.
